# Effectiveness and safety of traditional Chinese therapies intreating patients with amyotrophic lateral sclerosis: a protocol for systematic review and meta-analysis

**DOI:** 10.3389/fneur.2024.1519513

**Published:** 2024-12-31

**Authors:** Rui Li, Tingting Bao, Bei Li, Peng Xia, Tingting Zhang, Haoli Zhang, Fei Huang

**Affiliations:** ^1^Department of Acupuncture, Huangpi District Hospital of Traditional Chinese Medicine, Wuhan, Hubei, China; ^2^Emergency Department, Hubei Provincial Hospital of Traditional Chinese Medicine, Wuhan, China; ^3^Emergency Department, Affiliated Hospital of Hubei University of Chinese Medicine, Wuhan, China; ^4^Emergency Department, Hubei Institute of Traditional Chinese Medicine, Wuhan, China; ^5^School of Acupuncture and Tuina, Chengdu University of Traditional Chinese Medicine, Chengdu, China; ^6^Department of Tuina and Rehabilitation Medicine, Hubei Provincial Hospital of Traditional Chinese Medicine, Wuhan, China; ^7^Department of Tuina and Rehabilitation Medicine, Affiliated Hospital of Hubei University of Chinese Medicine, Wuhan, China; ^8^Department of Tuina and Rehabilitation Medicine, Hubei Institute of Traditional Chinese Medicine, Wuhan, China; ^9^Department of Rehabilitation, Ankang City Traditional Chinese Medicine Hospital, Ankang, China

**Keywords:** traditional Chinese therapies, amyotrophic lateral sclerosis, protocol, ALS, meta-analysis

## Abstract

**Background:**

Amyotrophic lateral sclerosis (ALS) is a chronic, progressive disease that affects both upper and lower motor neurons. Some physicians have used traditional Chinese therapies (TCT) to treat ALS. However, there has been no systematic review or meta-analysis to evaluate the effectiveness and safety of TCT interventions. This review aims to analyze the effects of TCT interventions for patients with amyotrophic lateral sclerosis.

**Methods and analysis:**

This study will include randomized, non-randomized, and quasi-experimental clinical trials, with participants being any age Amyotrophic Lateral Sclerosis (ALS) patients who have undergone TCT treatment. Two researchers will independently search databases including CENTRAL, PubMed, PEDro, EMBASE, CNKI, CBM, and SPORTDiscus, without restrictions on language or publication date. These researchers will independently screen titles and abstracts and extract data from the included studies. If deemed suitable for meta-analysis, data synthesis will be conducted using Review Manager V.5.3 software; any discrepancies will be resolved by a third researcher. The meta-analysis will compare the effects of TCT with placebo or other interventions. The main endpoint evaluated was the decrease in the overall score of the Amyotrophic Lateral Sclerosis Functional Rating Scale-Revised (ALSFRS-R; scoring from 0 to 48, where higher scores denote greater functionality) over a period of 24 weeks. Additional endpoints included the reduction rates in isometric muscle power, levels of phosphorylated axonal neurofilament H subunits in plasma, and slow vital capacity measurements. Furthermore, the study monitored the duration until occurrence of death, tracheostomy, or the need for long-term ventilation, as well as the time until death, tracheostomy, long-term ventilation, or hospital admission.

**Ethics and dissemination:**

Throughout the entire process of this systematic review, no personal information was used, hence ethical review is not required. The results of this meta-analysis will be disseminated through publication in peer-reviewed journals and/or conference presentations.

## Introduction

Amyotrophic Lateral Sclerosis (ALS), also known as Lou Gehrig’s disease, is a devastating neurodegenerative disorder characterized by the progressive degeneration and death of both upper and lower motor neurons (1). This leads to the loss of muscle control, resulting in weakness, paralysis, and ultimately respiratory failure. While medications such as riluzole, edaravone (Radicava), and tofersen (Qualsody) have been approved in the United States, and riluzole and tofersen are also available in Europe, these treatments do not cure the disease but primarily manage symptoms. In Japan, mecobalamin (Rozebalamin) has been approved, and South Korea has approved a stem cell therapy, although these approvals are based on limited data (2, 3). Additionally, microRNA therapy via adeno-associated virus (AAV) has been used in a few SOD1 mutation patients, with one patient showing improvement. Neurofilament light chain (NfL) is recognized as a biomarker in ALS disease progression and is used in clinical trials (4, 5). Despite extensive research efforts, treatment options for ALS remain limited. Current therapies can only moderately slow disease progression and alleviate some symptoms, leaving patients with limited treatment options and poor prognosis.

In recent years, there has been growing interest in Traditional Chinese Treatment (TCT) as an alternative or complementary therapy for ALS (6). TCT encompasses a rich history and holistic health approaches, including various interventions such as herbal medicine, acupuncture, and massage therapy. These treatments are based on the principle of restoring the balance of Qi and blood in the body, which is believed to be disrupted in the ALS process (7). However, the evidence base for the efficacy of TCT in ALS is currently limited, involving small patient populations and short study durations (8).

The effective mechanism of TCT treatment for ALS is still unclear. Although some clinical studies have shown positive results, the lack of standardized protocols and a comprehensive understanding of the underlying mechanisms have hindered the acceptance and integration of TCT into Western medical practice (8). This systematic review and meta-analysis aim to fill this gap by examining the efficacy and safety of various TCT treatments in ALS patients. By integrating data from multiple studies, we will evaluate the impact of (TCT) intervention on motor function, respiratory function, swallowing, and voice, providing a more reliable evidence base for its role in ALS treatment. This review will contribute to the growing evidence supporting the use of TCT in ALS management and provide valuable insights for clinicians and researchers in the field.

## Methods and analysis study registration

This systematic review protocol has been registered in the International Prospective Register of Systematic Reviews (PROSPERO) with the registration number CRD42023404689. All research steps will adhere to the guidelines of the Preferred Reporting Items for Systematic Reviews and Meta-Analyses (9).

### Patient and public involvement

In this study, no patients or other individuals were involved in the design, execution, reporting, or dissemination.

### Ethics and communication

This study is a systematic review. It does not collect private information from individual patients, and therefore does not require ethical review approval.

### Inclusion and exclusion criteria

#### Types of studies

All randomized, non-randomized, and quasi-experimental clinical trials will be included in the study, with no restrictions on publication time and language. Other types of studies, including reviews, animal experiments, theoretical discussions, case reports, conference papers, letters to the editor, and non-randomized controlled trials, will be excluded.

#### Types of participants

This study will include participants with ALS, regardless of age, occupation, and educational background. The diagnosis is based on the 2020 diagnostic criteria for Amyotrophic Lateral Sclerosis (Gold Coast criteria) recognized by the IFCN, WFN, ALS Association, and MND Association (10). Randomized, non-randomized, and quasi-experimental clinical trials of traditional Chinese medicine interventions in ALS patients will be evaluated. If crossover studies provide separate data for traditional Chinese medicine interventions (control and experimental), they will also be included.

#### Types of interventions

The intervention measures will include “Traditional Chinese Medicine” (Mesh term), acupuncture, herbal therapy, or massage therapy. All methods are derived from traditional Chinese medicine. Studies that are not related to the treatment of traditional Chinese medicine will be excluded.

#### Types of comparisons

The treatment method for the control group is not restricted. Therefore, the treatment for the control group can include no treatment at all, the use of a placebo, or any other control treatment deemed appropriate for comparison.

#### Types of outcome measures primary outcome

1. The primary outcomes will be the peripheral muscle strength assessed by manual muscle testing, muscle strength grading scales, and hand-held dynamometry.
2. Secondary outcomes will include function assessed by the Amyotrophic Lateral Sclerosis Function Score Scale-Revised Edition; Muscle activation as assessed by electromyography; Fatigue severity scale was used to evaluate the fatigue degree.

### Data sources and search strategies

Two independent reviewers (RL and TB) will conduct unrestricted searches of the following databases from their inception to October 1, 2022, to identify eligible publications: CENTRAL, Medline, PEDro, EMBASE, CNKI, CBM, and SPORTDiscus. The search strategy for the Medline (via PubMed) database was developed according to the Cochrane Handbook guidelines (see Table 1). The same search strategy will be used for other databases with appropriate adjustments for syntax. Before completing this review, these two reviewers will conduct another search to ensure the inclusion of the most recent studies.

**Table 1:** The search strategy for PubMed.

Search term	Search strategy
Subject headings	amyotrophic lateral sclerosis[Mesh]
Free text	amyotrophic lateral sclerosis OR ALS
Intervention terms	traditional chinese medicine”[Mesh] OR acupuncture OR herbal medicine OR massage therapy
Study types	randomized controlled trial[ptyp] OR non-randomized controlled trial[ptyp] OR quasi-experimental study[ptyp]
Language	No language restrictions to obtain as many relevant studies as possible.
Publication date	No publication date restrictions to include all relevant studies.

### Selection of studies

**Figure 1:**
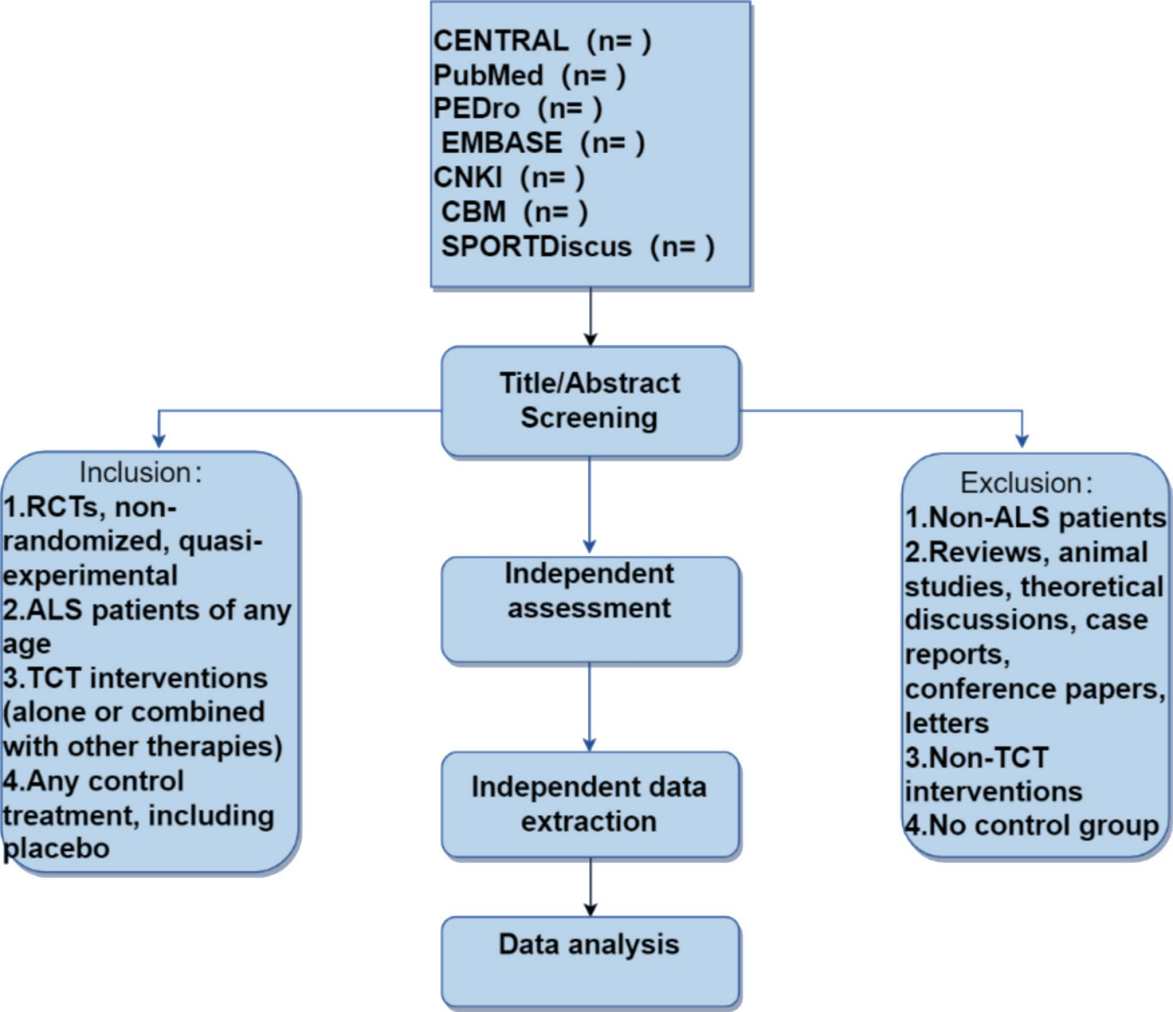
Flowchart of literature screening.

This systematic literature review will run from October 24, 2023, to May 10, 2024 (see Figure 1). All researchers have been trained to ensure they have a basic understanding of the background and purpose of this review. After the electronic search, relevant records will be uploaded to a database created by EndNote 21 software. Records selected from other sources will also be added to this database. Two researchers (BL and PX) will independently screen the titles, abstracts, and keywords of all retrieved studies and identify trials that meet the inclusion criteria. The full texts of all potentially relevant studies will be collected for further evaluation, and excluded studies will be documented with explanations. Any discrepancies will be resolved through discussion between the two researchers, and if necessary, a third researcher (TZ) will be involved in arbitration.

### Data extraction and management

All reviewers will collaboratively design a unified data extraction form. Then, two reviewers (RL and PX) will independently extract data in the following areas: basic information, participant characteristics, methods, interventions, outcomes, adverse events, and other relevant information. Any discrepancies will be discussed between these two reviewers, and if a consensus cannot be reached, a third author (TZ) will act as an arbitrator.

### Accounting for missing data

In handling missing data in the study, we will make every effort to contact the corresponding authors by email to supplement incomplete information. If the missing data cannot be filled, assumptions will be made according to the Cochrane guidelines for situations of “random missing” and “non-random missing” (11). For random missing data, only the existing data will be analyzed; in the case of non-random missing data, missing values will be estimated and the stability of the results will be tested through sensitivity analysis.

### Assessment of risk of bias

The potential for bias in the studies will be evaluated by two reviewers (HZ and FH) using a suitable tool from the Cochrane Collaboration. The bias risk in the selected studies will be assessed based on the following criteria: sequence generation; concealment of allocation; blinding of participants, staff, and outcome assessors; incomplete outcome data; selective outcome reporting; and other potential sources. The evaluations will be categorized as ‘low risk’, ‘high risk’, or ‘unclear risk’ (12).

### Data analysis and synthesis

Review Manager V.5.3 software will be utilized for data analysis and quantitative synthesis. For continuous data, the standardized mean differences with 95% confidence intervals will be employed to evaluate treatment effects. For dichotomous data, risk ratios with 95% confidence intervals will be applied. Depending on data heterogeneity, either a random effects model (I2 < 50%) or a fixed effects model (I2 ≥ 50%) will be used for analysis. Furthermore, if heterogeneity is deemed significant, subgroup or sensitivity analyses will be conducted to pinpoint its origin. In cases where data is inadequate for quantitative analysis, a descriptive analysis will be performed.

#### Subgroup analysis

If the results show high heterogeneity, sub-group analyses will be conducted based on the type of TCT intervention, the severity of muscle strength loss, the duration of treatment, and other relevant parameters.

#### Sensitivity analysis

A sensitivity analysis will be conducted by reassessing methodological quality, study type, sample size, missing data, and other potential factors to confirm the reliability of the primary findings. Should a significant discrepancy arise, the cause of the difference will be rigorously examined.

### Assessment of reporting biases

If more than eight study reports measure the same results, we will use funnel plots and Egger’s regression test to assess reporting bias.

### Grading the quality of evidence

The Grading of Recommendations Assessment, Development and Evaluation (GRADE) working group’s methodology will be employed to evaluate the quality of evidence for all outcomes (13). The assessment will encompass six domains: risk of bias, consistency, directness, precision, publication bias, and additional points. These evaluations will be categorized as ‘high’, ‘moderate’, ‘low’, or ‘very low’.

## Discussion

TCT is a medical system with a profound history and a rich theoretical framework, the origins of which can be traced back to the Shang Dynasty (1766–1,122 BC) (14). With the migration of Chinese people and the spread of culture, TCT has gradually spread to East Asia and other regions. As global attention turns to traditional medicine, many countries have begun to value the research on TCT, especially in Europe and America (15). An increasing number of research institutions and universities have established research centers for TCT, conducting basic research and clinical trials (16). These studies not only enrich the clinical applications of TCT but also provide a scientific basis for its international acceptance. To evaluate the actual effects of TCT in ALS patients, researchers have developed the ALS-SSIT (Amyotrophic Lateral Sclerosis Synthetic TCT Efficacy Evaluation Tool), which is considered a feasible, reliable, and sensitive evaluation method (17). With this tool, clinicians can better monitor patients’ changes during the process of integrated Chinese and Western medicine treatment. In addition to protocols based on traditional Chinese medicine, there are other specific protocols available for the diagnosis and management of ALS. Research results show that in the early stages of the disease, patients’ oral and pharyngeal structures are affected, manifesting as voice changes and difficulty swallowing. Multidisciplinary teamwork, including otolaryngology, is undoubtedly very important in the diagnosis and treatment of bulbar muscular atrophy ALS (18). Additionally, studies have found that acoustic analysis and pronunciation analysis help identify early signs of bulbar muscular atrophy ALS and assist in identifying changes in voice parameters for early detection and prediction of the involvement and disease progression of bulbar muscular atrophy ALS, as well as targeted intervention (19).

Riluzole and edaravone have achieved certain positive effects in the treatment of Amyotrophic Lateral Sclerosis (ALS), although these effects may not be significant (10, 20). Riluzole was the first drug approved by the U.S. Food and Drug Administration (FDA) for the treatment of ALS. Its mechanism of action is not fully understood but is speculated to reduce glutamatergic neurotransmission by blocking voltage-gated sodium channels on the presynaptic neuronal membrane. Riluzole is relatively safe, although the most common side effects include elevated liver enzyme levels and fatigue (21). Edaravone is considered to slow disease progression in highly selective early-onset and rapidly progressing ALS patients. It has been approved by the FDA but has not yet received approval from the European Medicines Agency (EMA) (22). However, whether edaravone should be provided to all ALS patients regardless of clinical presentation remains controversial. Tofersen, a muscle relaxant, has shown effects of slowing disease progression in some patients and improving function in others in a phase II randomized controlled trial in patients with ALS. These preliminary research results bring new hope to the field of ALS treatment, but more research is needed to confirm these findings and further explore their potential therapeutic mechanisms (20).

In recent medical research, the treatment of ALS has received increasing attention. The combination of TCT and Modern medicine, especially in the context of new drugs combined with TCT, has shown a promising future. Multiple clinical studies are exploring the effectiveness of integrated TCT and Modern medicine in the treatment of ALS. For example, clinical trials of integrated TCT and Modern medicine have shown that the treatment group combining TCT and Modern medicine has more significant improvements in functional outcomes and quality of life compared to Modern medicine alone. In these studies, patients receiving standard Modern medicine treatment also used specific Chinese herbal formulas, and the results showed significant improvements in muscle strength and motor ability. As ALS is such a novel disease, it is necessary to analyze the efficacy and safety of TCT as a treatment. Our research may provide evidence for evaluating TCT as a treatment for ALS. These results may provide insights for clinicians treating ALS and can serve as guidelines for TCT (23).

## Strengths and limitations of this study

1. This study employed an extensive search strategy, covering seven databases and including English and Chinese language sources, which increased the likelihood of identifying all relevant studies on TCT interventions for ALS.
2. Inclusion of diverse study designs: by incorporating randomized, non-randomized, and quasi-experimental clinical trials, this review offers a more comprehensive assessment of the efficacy and safety of TCT interventions.
3. Independent data extraction and analysis: utilizing two independent researchers for data screening, extraction, and synthesis minimized the risk of bias and ensured the reliability of the findings.
4. Publication bias: there is a risk of publication bias, as studies with negative or non-significant results may be less likely to be published. This could affect the overall conclusions of the meta-analysis.
5. Quality of included studies: the inclusion of non-randomized and quasi-experimental studies may introduce methodological limitations, which could impact the validity of the findings.
6. Heterogeneity of interventions: TCT interventions can vary widely, making it challenging to compare and synthesize the results across different studies.
